# Andean drought and glacial retreat tied to Greenland warming during the last glacial period

**DOI:** 10.1038/s41467-020-19000-8

**Published:** 2020-10-12

**Authors:** Arielle Woods, Donald T. Rodbell, Mark B. Abbott, Robert G. Hatfield, Christine Y. Chen, Sophie B. Lehmann, David McGee, Nicholas C. Weidhaas, Pedro M. Tapia, Blas L. Valero-Garcés, Mark B. Bush, Joseph S. Stoner

**Affiliations:** 1 Department of Geology and Environmental Science, University of Pittsburgh, Pittsburgh, PA USA; 2 Geology Department, Union College, Schenectady, NY USA; 3 College of Earth, Ocean, and Atmospheric Science, Oregon State University, Corvallis, OR USA; 4 Department of Geological Sciences, University of Florida, Gainesville, FL USA; 5 Department of Earth, Atmospheric, and Planetary Sciences, Massachusetts Institute of Technology, Cambridge, MA USA; 6 Division of Geological and Planetary Sciences, California Institute of Technology, Pasadena, CA USA; 7 Instituto Nacional de Investigación en Glaciares y Ecosistemas de Montaña, Ancash, Peru; 8 Pyrenean Institute of Ecology, Spanish National Research Council, Zaragoza, Spain; 9 Florida Institute of Technology, Melbourne, FL USA

**Keywords:** Atmospheric science, Palaeoclimate, Limnology

## Abstract

Abrupt warming events recorded in Greenland ice cores known as Dansgaard-Oeschger (DO) interstadials are linked to changes in tropical circulation during the last glacial cycle. Corresponding variations in South American summer monsoon (SASM) strength are documented, most commonly, in isotopic records from speleothems, but less is known about how these changes affected precipitation and Andean glacier mass balance. Here we present a sediment record spanning the last ~50 ka from Lake Junín (Peru) in the tropical Andes that has sufficient chronologic precision to document abrupt climatic events on a centennial-millennial time scale. DO events involved the near-complete disappearance of glaciers below 4700 masl in the eastern Andean cordillera and major reductions in the level of Peru’s second largest lake. Our results reveal the magnitude of the hydroclimatic disruptions in the highest reaches of the Amazon Basin that were caused by a weakening of the SASM during abrupt arctic warming. Accentuated warming in the Arctic could lead to significant reductions in the precipitation-evaporation balance of the southern tropical Andes with deleterious effects on this densely populated region of South America.

## Abstract (web summary)

How the abrupt warming events recorded in Greenland ice cores during the last glacial cycle have influenced the tropical climate is not well known. Here the authors present new lake sediment data from the Peruvian Andes that shows that these events resulted in rapid glacier retreat and large reductions in lake level.

## Introduction

Variations in Atlantic Meridional Overturning Circulation (AMOC) during the last glacial cycle drove abrupt changes in the thermal gradient of the North Atlantic sector, altering the interhemispheric distribution of tropical heat, the mean position of the intertropical convergence zone (ITCZ), and trade wind strength^1–3^. South American low-latitude paleoclimate proxy records are sensitive to high-latitude forcing via the strength of the South American summer monsoon (SASM), which increased during cold stadial periods such as Heinrich events^4–7^, and weakened during the abrupt warmings recorded in Greenland ice cores associated with Dansgaard–Oeschger (DO) interstadials^5,8,9^.

Large fluctuations in Andean paleolake levels have been documented on the Bolivian Altiplano in association with the Younger Dryas and Heinrich events 1 and 2^7,10,11^, and Andean glacier advances or stillstands have been linked to these wet events in some cases^12^. Less is known about the effects of the shorter duration DO cycles on precipitation anomalies and on the mass balance of tropical Andean glaciers. Although some studies proposed a causal link between local glacier fluctuations and the sedimentary record of Lake Titicaca^13^, well-dated continuous records are necessary to test this hypothesis. Much of the paleoclimatic evidence documenting these rapid changes in tropical South American hydroclimate relies on the interpretation of δ^18^O variations in speleothems from the Amazon Basin and surrounding regions^5,6,8^. Similarities among speleothem δ^18^O records reflect the regional impact of variations in convective activity and upstream rainout in the core monsoon region of Amazonia^14^. However, speleothem records from several localities do not reveal a tight coupling between independent proxies of local precipitation amount and the δ^18^O of that precipitation (δ^18^O_precip_)^15,16^, indicating that upstream factors other than the amount effect^17^ may dominate δ^18^O_precip_ at some locations. The inability to isolate local precipitation variations from the composite δ^18^O signal^18,19^ makes it difficult to assess the specific impact of abrupt Arctic warming on water availability and glacial mass balance in the tropical Andes, and it highlights the need for δ^18^O-independent records of hydroclimate.

**Fig. 1:**
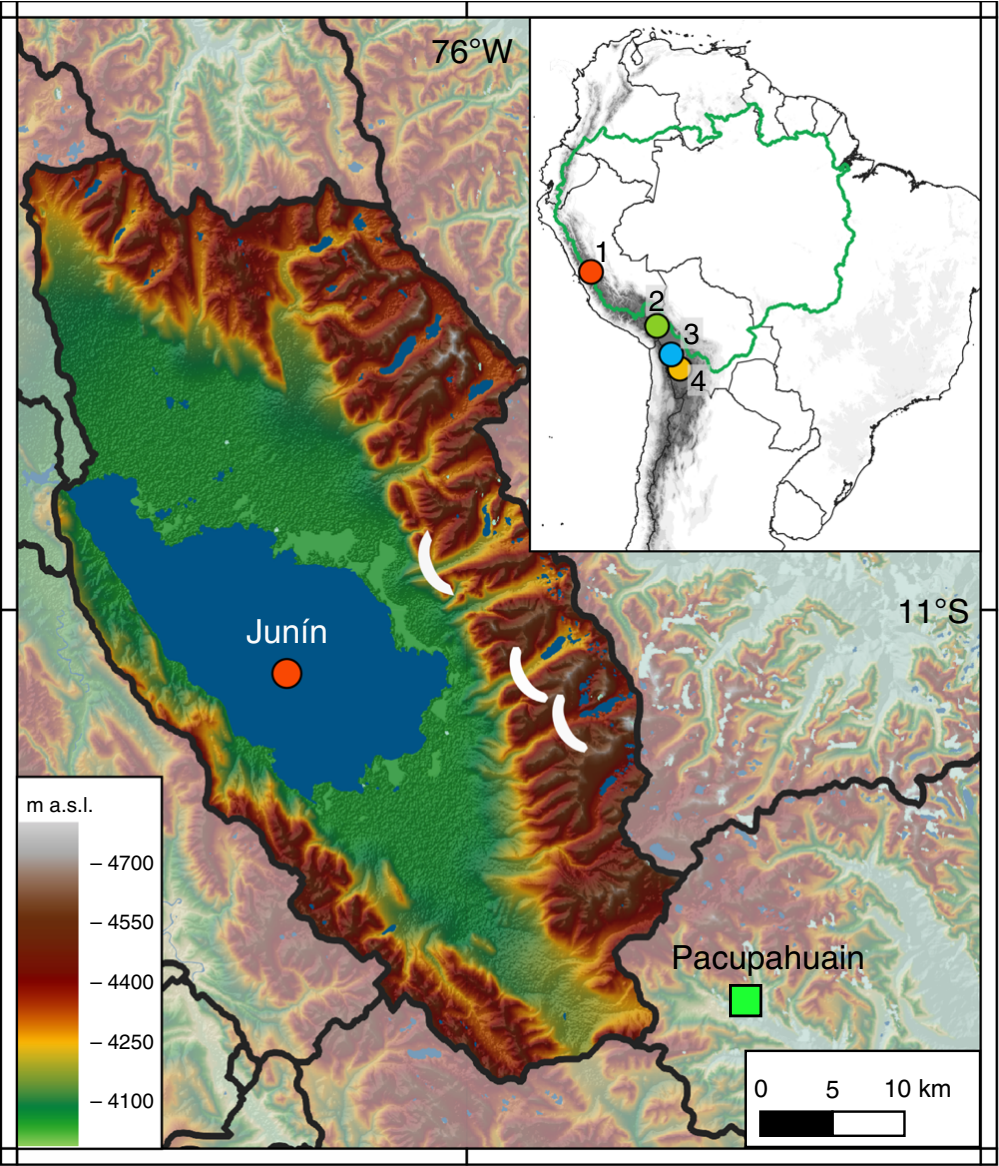
Location of the Lake Junín (4100 masl) drainage basin and Pacupahuain cave in central Peru. White ellipses in valleys east of Lake Junín indicate the mapped down valley extent of glaciers during the local Last Glacial Maximum^24^. Inset map: green line indicates the Amazon drainage basin, and circles indicate Andean records discussed in the text: 1. Junín, 2. Lake Titicaca^13^, 3. Paleolake Tauca^10,12^, 4. Salar de Uyuni^7^.

Here we present a sediment record from a high elevation lake in the central Peruvian Andes that records lake level fluctuations as well as changes in paleoglacier mass balance. We show that the DO interstadials between 50 and 15 ka, which are recorded isotopically both in Greenland ice^20,21^ and speleothem δ^18^O from Pacupahuain Cave in the upper Amazon Basin^5^, were associated with rapid and large reductions in Andean precipitation amount recorded by multiple independent proxies in Lake Junín sediments. Many of these perturbations were sufficient to deglaciate the adjacent portion of the eastern Andean cordillera up to at least 4700 masl and profoundly shrink Lake Junín, Peru’s second-largest lake located at 4100 masl and ~25 km from Pacupahuain Cave (Fig. 1). This record documents the unambiguous impact on glacier mass balance and hydroclimate of the climatic teleconnection linking the Atlantic meridional thermal gradient with the strength of the SASM over the past 50 ka.

## Results

### Lake setting and glacial connection

Lake Junín (11°S) is a seasonally closed-basin lake located between the eastern and western cordilleras of the central Peruvian Andes (Fig. 1). With a surface area of ~280 km^2^ and a seasonally variable water depth of ~8–12 m, Lake Junín is especially sensitive to changes in precipitation–evaporation balance (P–E). The watershed occupies the Puna grasslands ecoregion where groundwater-fed peatlands (*bofedales*), characterized by organic-rich sediment, occupy the shallow water lake margins. Glacial outwash fans and lateral moraines form the basin’s eastern and northern edges (Fig. 1), and ^10^Be exposure ages from these moraines indicate they span multiple glacial cycles^22,23^, but at no time during at least the last 50 ka has the lake been overridden by glacial ice^24^. Thus, Lake Junín is ideally situated to record the last glacial cycle in the adjacent eastern cordillera. During the local last glacial maximum (LLGM; ~28.5–22.5 ka) alpine glaciers descended from headwall elevations as high as ~4700 masl to end moraine positions ~4160 masl, within several km of the modern shoreline^24^. Whereas glaciers in the inner tropics of the Andes are especially temperature-sensitive because of sustained precipitation year-round, glaciers in the outer tropics, such as those at the latitude of the Junín basin, experience greater seasonality of precipitation and are twice as sensitive to changes in precipitation as those in the inner tropics^25,26^. The Junín region receives most of its moisture through the SASM during the austral summer (DJF) with <7% falling during the winter (JJA), making variations in the SASM a principal driver of changes in paleoglacier mass balance.

Most records of glaciation in the tropical Andes rely on moraine exposure ages to infer the timing and extent of advances^12,23,27^. However, such records are limited by age uncertainties of ~±5%, an unknown temporal relationship between the timing of moraine stabilization and ice advance, and the tendency for larger advances to erase evidence of prior glacial cycles. Continuous proxy records from well-dated glacier-fed lakes such as Junín can compensate for such limitations, with clastic sediment flux and high-resolution XRF scans being well-established proxies for glacial erosion of bedrock that, in turn, reflect relative changes in paleoglacier activity and mass balance^28,29^. Accordingly, final deglaciation of the Junín watershed was nearly complete by 18 ka and was marked by a near-total cessation of clastic sediment input to the lake^29,30^.

### Sedimentary context

**Fig. 2:**
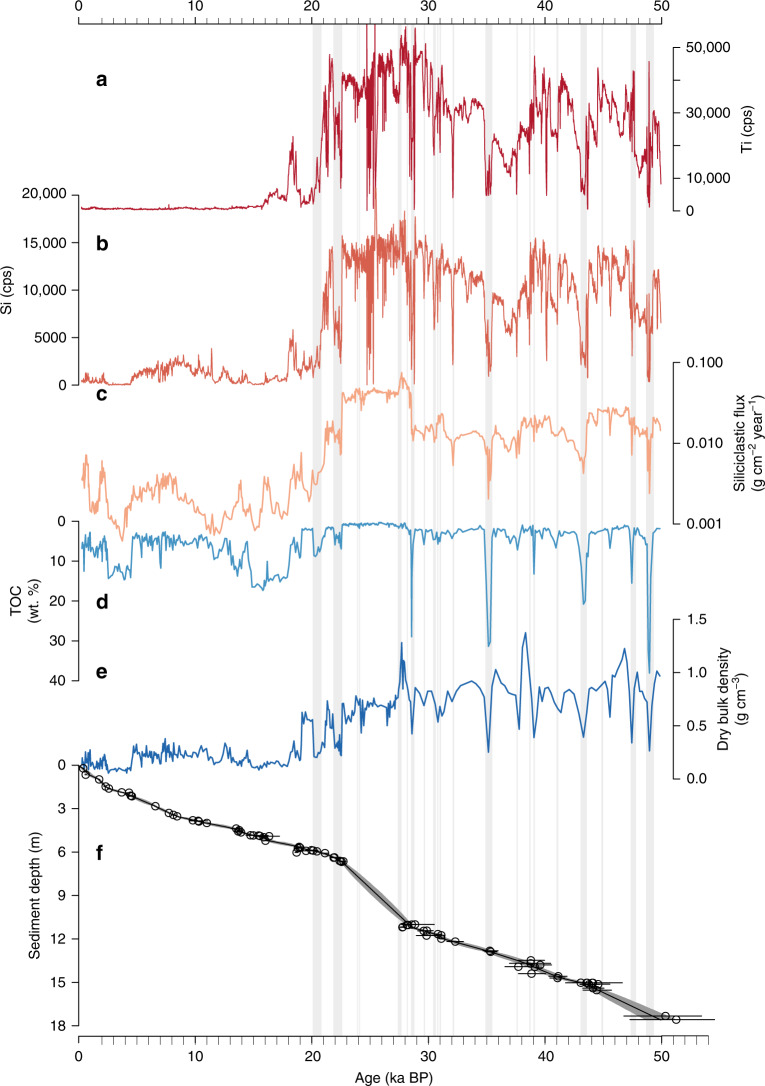
Physical and geochemical sediment properties from the Junín drill core. The similar XRF profiles of **a** Ti and **b** Si indicate both elements primarily represent clastic inputs, with slight differences attributable to different bedrock mineralogy and grain size. **c** Siliciclastic sediment flux (log scale). **d** Total organic carbon (TOC). **e** Dry bulk density. **f** Bacon age-depth model of 79 AMS radiocarbon ages on terrestrial macrofossils; error bars denote 95% probability range for calibrated ages. Ages excluded from the age model are listed in the supplemental. Gray vertical bars show the distribution of peat layers.

The Junín sediment cores were obtained from the lake depocenter (Fig. 1) in 8.2 m of water. The age model for the last 50 ka (cal yr BP, 1950 CE) is based on 79 radiocarbon measurements from terrestrial macrofossils and charcoal (Fig. 2f and Supplementary Table 1). Sediment deposited from 50–22.5 ka is dominated by fine-grained glacial flour characterized by high Ti and Si counts per second (cps), high density, and low total organic carbon (TOC) (Fig. 2a–e). Glacigenic sediment input to Lake Junín was especially high and devoid of plant macrofossils from 28.5–22.5 ka (Fig. 2a–c), which corresponds to the age of moraines deposited during the maximum extent of ice in the last 50 ka in the adjacent eastern cordillera^23^, according to ^10^Be ages recalculated based on updated production rates and scaling factors^31^. The scaling procedure applied here^31^ yields ^10^Be ages that are compatible within ±2% with those that would be obtained using local tropical Andes ^10^Be production rates^32,33^. Glacigenic sediment deposition was punctuated by a series of distinct 1–20-cm-thick peat layers (Fig. 2) containing 5–35% TOC (Fig. 2d) with abundant macrofossils that are similar to the sediment accumulating today in the fringing peatlands. These peat layers span intervals from ~25–500 years based on mean sedimentation rates and are interpreted to reflect lake low stands when surrounding peatlands encroached toward the center of the lake, forming a record of repeated water level fluctuations of up to ±8 m. There is no sedimentary or radiocarbon evidence (Fig. 2f) for unconformities within age model precision, which indicates that while these peat layers represent considerably lower water level, the drill site remained submerged, at least seasonally, for the duration of our record. Today Lake Junín overflows during the summer wet season and there is no evidence of shoreline features above the modern lake level. Sediment deposited after ~20 ka reveals a rapid decline in clastic input, evidenced by the low values of Ti, Si, bulk density, and siliciclastic flux. The near-complete loss of the clastic signal does not imply reduced precipitation after 20 ka, but rather reflects a different sedimentary environment wherein glaciers had retreated behind up valley moraine-dammed lakes that served as local sediment traps. During the late glacial, the sedimentary regime shifted to a lake increasingly dominated by authigenic CaCO_3_ with frequent laminations (Fig. 2c–e and Supplementary Fig. 1). The occasional fine-grained organic-rich intervals <20 ka contain few plant macrofossils and record autochthonous (algal) productivity; these intervals do not resemble the dark, crumbly, and macrofossil-rich peats that punctuate the 20–50 ka interval of the core.

### Synchronous changes in lake level and paleoglacier extent

**Fig. 3:**
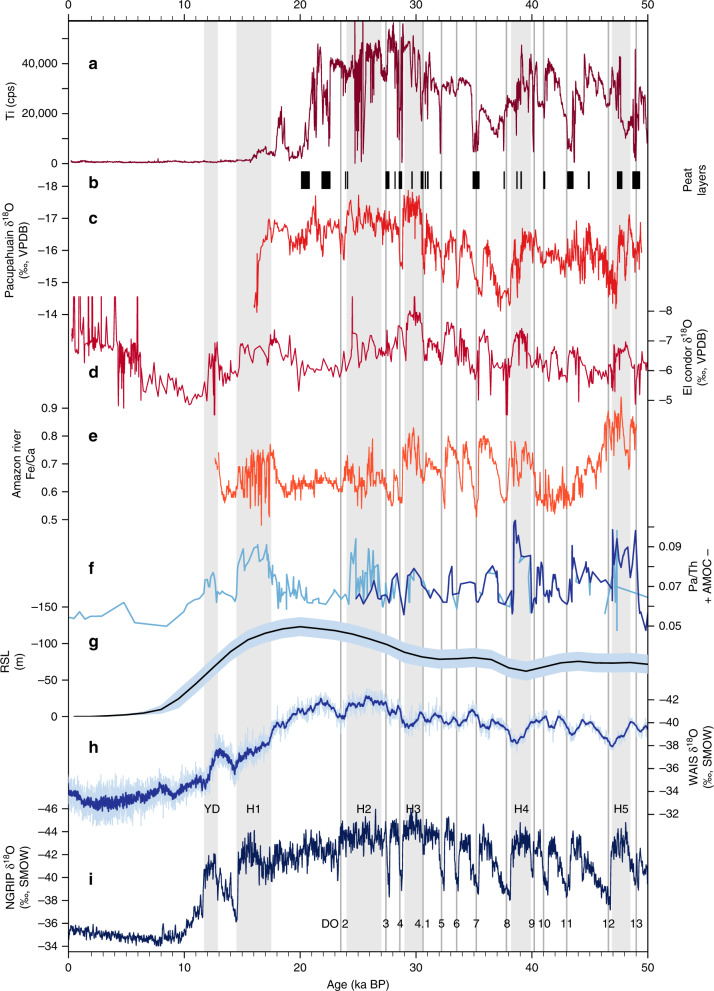
Comparison of regional and global proxy paleoclimatic records. **a** Junín glaciation (Ti from Fig. 2a). **b** Junín low stands (peat layers). **c** Pacupahuain speleothem δ^18^O^5^. **d** El Condor speleothem δ^18^O^8^. **e** Amazon Discharge^9^. **f** Atlantic Meridional Overturning Circulation (AMOC) strength (dark blue curve is Pa/Th data reported in (1)), and the light blue curve is a compilation of previously reported Pa/Th records as presented in (1). **g** Relative sea level^37^. **h** WAIS Divide ice core δ^18^O^38^. **i** NGRIP ice core δ^18^O^20,21^. Vertical gray boxes denote the Younger Dryas and Heinrich stadials H1–H5, and numbered vertical lines are Dansgaard–Oeschger (DO) warming events 2–13.

The Junín record exhibits a reduced input of glacigenic sediment during DO interstadials 4–13 (Fig. 3a), with all but two of these intervals marked by enhanced peat accumulation, associated higher TOC, and lower density (Fig. 2d, e). Similar but less pronounced changes appear to be associated with DO interstadials 2 and 3. However, the timing of these latter events coincides with the period of rapid clastic sedimentation during the LLGM, an interval that is devoid of datable macrofossils and therefore has less robust age control (Fig. 2f). Declines in siliciclastic sediment flux (Fig. 2c) indicate that simple dilution effects were not responsible for the reductions in glacigenic sediment concentration. The timing of DO interstadials was thus marked by widespread glacial retreat and lake level lowering up to ~8 m, within the chronologic uncertainty of our age model (Supplementary Fig. 2). The absence of evidence for lowered lake level during complete and final deglaciation of the Junín catchment (~20–15 ka), when regional warming drove snowlines to rise 300–600 m^24,30^, indicates that the level of Lake Junín is especially sensitive to precipitation amount rather than variations in temperature. The close association between low lake stands and reduced glacial sediment flux during DO events suggests that these reductions in paleoglacier mass balance were primarily driven by decreases in precipitation. The declines in lake level associated with the DO events noted here corroborates the evidence of water level reductions associated with DO interstadials 11, 10, and 8 at 1360 masl in southern Peru (14°S)^34^, and evidence for millennial-scale fluctuations in nearshore terrigenous inputs to Lake Titicaca that may be linked to DO events^13^. The documented changes in hydroclimate in the Junín region may thus have affected a large region of the westernmost Amazon Basin, which is consistent with the Fe/Ca record of Amazon River discharge^9^ (Fig. 3e). Relative to Altiplano lake records^7,10^, Junín most strongly records dry (DO) events, whereas the Altiplano records are more sensitive to wet (Heinrich) events. Thus, the records appear to be biased toward recording hydroclimatic perturbations that contrast the most with their respective background states; namely, more arid on the Altiplano and more humid in the Junín region.

## Discussion

On millennial timescales, multiple independent proxies measured on Junín sediments bear a strong correspondence to the precisely dated speleothem δ^18^O records from both the nearby Pacupahuain Cave^5^ (Fig. 3c) and from El Condor Cave (Fig. 3d), a lower elevation site (800 masl) in the western Amazon Basin of northern Peru^8^. This concurrence indicates that regional monsoon strength was a first-order control on all records. While downcore changes in clastic sediment inputs at Junín are indicative of relative, rather than absolute, changes in precipitation, they record an entirely local signal. The observed similarity with nearby speleothems suggests that δ^18^O_precip_, which has been interpreted to reflect upstream convection and rainout^5,14,35^, also reflects some degree of variable local precipitation amount in the tropical Andes. However, the magnitude and duration of Junín’s response to individual DO events is often not to scale with that of Pacupahuain, only 25 km away. For example, DO interstadials 11 and 13 register as profoundly dry intervals at Junín but only minimally so in Pacupahuain, contrary to the signal that would be predicted by a simple amount effect^17^. A similar mismatch occurs during DO interstadial 8, which is a relatively weak dry period at Junín with moderate reductions in Ti and Si and only a multi-decadal interval of peat accumulation, yet DO 8 in the Pacupahuain record is marked by the most positive δ^18^O excursion in the entire speleothem sequence, lasting nearly a millennium. These observations indicate that the local moisture response at Junín can be disproportional to, and possibly even decoupled from, the δ^18^O signal that is thought to be recording millennial-scale SASM intensity. This confirms earlier work showing that atmospheric transport of water vapor from the tropical Atlantic across the Amazon lowlands involves numerous isotopic controls, in addition to precipitation amount, which influences the δ^18^O_precip_ signal of geologic archives^18,19,35^.

The early onset of deglaciation in the central Peruvian Andes, ~22.5 ka based on lake sediment records^36^ (Fig. 3a), is consistent with moraine ages that reflect retreating ice margins at this time^23,27^. This onset was several millennia prior to the onset of global deglaciation as recorded by sea-level rise^37^ (Fig. 3g), and was initially interpreted as evidence for early tropical warming because of the lack of evidence for drying at this time^36^. The Junín peat record, however, reveals that two prolonged droughts, lasting a total of ~1300 yr, occurred in quick succession (22.5–21.9 ka and 20.8–20.1 ka), just prior to the onset of warming ~20 ka in the high latitudes of the Southern Hemisphere^38^ (Fig. 3h). We suggest that these prolonged dry intervals, which resemble Junín’s response to DO events, were responsible for the early onset of glacial retreat in this region of the tropical Andes. These abrupt reductions in precipitation at Junín are evident, though subtle, in the Pacupahuain record, yet they do not appear as pronounced individual excursions in AMOC^1^ or Amazon discharge^9^ (Fig. 3e, f). It is notable, however, that the latter two records indicate that the period from ~24 to 19 ka was characterized by a relatively strong AMOC and overall drier conditions in the Amazon Basin, respectively. These observations, along with records of tropical Atlantic mixed layer depth^3^, indicate that the 24–19 ka interval was not marked by the large southward ITCZ displacements that characterized Heinrich events 2 and 1, and this may explain why Junín experienced extended droughts and early deglaciation during this interval. Alternately, modeling studies have pointed to a thermodynamically driven contraction of the tropical rain belt associated with global cooling during the global LGM^39^, which may have contributed to reductions in SASM rainfall and early deglaciation in the tropical Andes.

The significant disruption to glaciers and hydroclimate in the tropical Andes in response to perturbations in the meridional temperature gradient of the North Atlantic documented here demonstrates the sensitivity of tropical P–E balance to the Northern Hemisphere climatic shifts. There are multiple possible scenarios for regional hydroclimatic change in the Amazon Basin in response to twenty-first-century warming. One scenario posits that accentuated warming in the Arctic will result in a northward shift in the mean position of the ITCZ^40,41^, while another projects a stable mean position of the ITCZ, but reductions in both width and strength^42^. Past changes in the hemispheric thermal gradient are an imperfect analog for future change, however. For example, the attendant increase in AMOC strength documented during warm interstadials is not expected to accompany future CO_2_-induced warming^43^, and changes in ITCZ position could be superimposed^41^ on enhanced tropical precipitation that is projected by some models^44^. Nevertheless, a northward shift in the thermal equator remains likely given faster heating of the Northern Hemisphere, and this scenario is consistent with a multimodel ensemble of simulations which projects regional drying of Amazonia and much of the tropics^45^. This would lead to significant reductions in P–E in the tropical Andes with impacts on glaciers, water supplies, hydropower, and agriculture in a region inhabited by tens of millions of people.

## Methods

### Chronology

Samples and standards for radiocarbon age determination were chemically pretreated, vacuum sealed, and combusted at the University of Pittsburgh according to standard protocols (https://sites.uci.edu/keckams/files/2016/12/aba_protocol.pdf), and graphitized and dated by accelerator mass spectrometry at the W.M. Keck Carbon Cycle AMS facility at the University of California, Irvine. Radiocarbon samples obtained from off-splice core sections were assigned stratigraphically equivalent splice depths; the development of the splice is detailed elsewhere^46^. Radiocarbon age measurements with errors and median calibrated age estimates are reported in Table S1. An age model was constructed based on 79 radiocarbon ages spanning the upper 17.6 m of sediment. The IntCal 13 calibration curve^47^ was used to calibrate all dates <50,000 cal yr BP, and the CalPal2007_Hulu calibration curve^48^ was used for those >50,000 cal yr BP. Bayesian age-depth modeling was performed using the R software package Bacon v. 2.3^49^ with the following settings: acc.mean = 25 yr cm^−1^, acc.shape = 1.5, mem.strengh = 4, mem.mean = 0.7, and thick = 5 cm.

Initial age model test runs indicated that in some instances the mean age estimates were biased toward outliers at the expense of clusters of more consistent dates. Therefore, dates that fell outside of the model’s 95% confidence interval were excluded prior to final age model construction if they also met other criteria, such as small sample mass (<1 mg) or low CO_2_ gas yield (<10 Torr) when a larger sample was available from the same interval, or if a reversal was evident based on multiple surrounding dates. In addition, the two oldest measured dates that are within the IntCal 13 calibration range (UCIAMS# 193160, 193169) would imply that sedimentation rates from ~45–50 ka were higher than any other portion of the record, including the LGM. We suggest these two ages are erroneously young and warrant exclusion. The stratigraphy does not indicate a change in sedimentation regime during this interval, and there is a pair of older dates between them (calibrated using the CalPal2007_Hulu curve) that are in close stratigraphic and chronologic agreement. Finally, sample 201045 yielded the largest lab error and is closest to the limits of the radiocarbon method, and it represents an age-reversal of several thousand years in the context of three underlying samples. Dates excluded from the final age model are marked by an asterisk in Supplementary Table 1. Calibrated median ages and 95% probability ranges are rounded to the nearest 5 yr for ages <1000, the nearest 10 yr for 1000–10,000, the nearest 50 yr for 10,000–20,000, and the nearest 100 yr for >20,000.

### Physical analysis of sediment cores

Bulk density was determined from the air-dried mass of 1 cm^3^ sample taken every 2.5 cm above 6.665 m and every 8 cm below 6.665 m. Total organic and inorganic carbon (TOC, TIC) was measured on samples taken every 2.5 cm above 6.665 m and every 4 cm below 6.665 m. Total carbon (TC) was determined by combusting samples at 1000 °C using a UIC 5200 automated furnace, and analyzing the resultant CO_2_ using a UIC 5014 coulometer at Union College. Similarly, TIC was determined by acidifying samples using an Automate acidification module and measuring the resultant CO_2_ by coulometry at Union College. We calculated the weight percentage TOC from TOC = TC − TIC. We measured the biogenic silica (bSiO_2_) content of a total of 65 samples obtained randomly from all facies present in the sediment core. Siliciclastic flux (Flux_clastic_, Eq. 1) was calculated as:

1 $${\mathrm{Flux}}_{{\mathrm{clastic}}} = {\mathrm{SR}} \ast \left( {{\mathrm{BD}} - \left( {\left( {{\mathrm{BD}} \ast {\mathrm{TOM}}} \right) + \left( {{\mathrm{BD}} \ast {\mathrm{TCC}}} \right)} \right)} \right),$$

where SR is bulk sedimentation rate (cm yr^−1^), BD is bulk density (gm cm^−3^), TOM is the weight fraction organic matter of the bulk sediment, and TCC is the weight fraction CaCO_3_ of the bulk sediment. We calculated TOM from TOC (%)/44 to reflect the molar ratio between plant cellulose (C_6_H_10_O_5_)*n* and TOC (%), and we calculated TCC from TIC (%)/12 to reflect the molar ratio between TIC (%) and CaCO_3_. Because of the presence of both authigenic and detrital CaCO_3_ in the sediment core, the removal of all CaCO_3_ in the estimation of Flux_clastic_ results in an underestimation of the total detrital flux during intervals of high clastic input, and this, in turn, reduces the amplitude of change in clastic flux between intervals of high and low glacigenic sediment input. While we do not explicitly remove the bSiO_2_ in the calculation of Flux_clastic_, the average weight percentage bSiO_2_ for all 65 samples facies samples is 0.92 ± 1.12% (±1σ), and thus is negligible.

XRF scanning was performed at the LacCore XRF Lab, University of Minnesota-Duluth Large Lakes Observatory using a Cox Analytical ITRAX with a Cr tube, 5 mm resolution, and 15 s dwell time.

## Supplementary information

Supplementary Information

Peer Review File

## Data Availability

All data sets generated during the current study are available on the NOAA World Data Service for the Paleoclimatology archive at https://www.ncdc.noaa.gov/paleo/study/31152.

## References

[1] Henry LG (2016). North Atlantic ocean circulation and abrupt climate change during the last glaciation. Science.

[2] McGee D (2018). Hemispherically asymmetric trade wind changes as signatures of past ITCZ shifts. Quat. Sci. Rev..

[3] Portilho-Ramos RC (2017). Coupling of equatorial Atlantic surface stratification to glacial shifts in the tropical rainbelt. Sci. Rep..

[4] Arz HW, Pätzold J, Wefer G (1998). Correlated millennial-scale changes in surface hydrography and terrigenous sediment yield inferred from last-glacial marine deposits off Northeastern Brazil. Quat. res..

[5] Kanner LC, Burns SJ, Cheng H, Edwards RL (2012). High-latitude forcing of the South American summer monsoon during the last glacial. Science.

[6] Wang X (2004). Wet periods in northeastern Brazil over the past 210 kyr linked to distant climate anomalies. Nature.

[7] Baker PA (2001). Tropical climate changes at millennial and orbital timescales on the Bolivian Altiplano. Nature.

[8] Cheng H (2013). Climate change patterns in Amazonia and biodiversity. Nat. Commun..

[9] Zhang Y (2017). Different precipitation patterns across tropical South America during Heinrich and Dansgaard-Oeschger stadials. Quat. Sci. Rev..

[10] Placzek C, Quade J, Patchett PJ (2006). Geochronology and stratigraphy of late Pleistocene lake cycles on the southern Bolivian Altiplano: implications for causes of tropical climate change. Geol. Soc. Am. Bull..

[11] Blard P-H (2011). Lake highstands on the Altiplano (Tropical Andes) contemporaneous with Heinrich 1 and the Younger Dryas: new insights from 14C, U–Th dating and δ18O of carbonates. Quat. Sci. Rev..

[12] Martin LCP (2018). Lake Tauca highstand (Heinrich Stadial 1a) driven by a southward shift of the Bolivian High. Sci. Adv..

[13] Fritz SC, Baker PA, Ekdahl E, Seltzer GO, Stevens LR (2010). Millennial-scale climate variability during the Last Glacial period in the tropical Andes. Quat. Sci. Rev..

[14] Vuille M, Werner M (2005). Stable isotopes in precipitation recording South American summer monsoon and ENSO variability: observations and model results. Clim. Dyn..

[15] Ward BM (2019). Reconstruction of Holocene coupling between the South American Monsoon System and local moisture variability from speleothem δ18O and 87Sr/86Sr records. Quat. Sci. Rev..

[16] Wortham BE (2017). Assessing response of local moisture conditions in central Brazil to variability in regional monsoon intensity using speleothem 87Sr/86Sr values. Earth Planet. Sci. Lett..

[17] Dansgaard W (1964). Stable isotopes in precipitation. Tellus.

[18] Lee J-E, Johnson K, Fung I (2009). Precipitation over South America during the Last Glacial Maximum: an analysis of the “amount effect” with a water isotope-enabled general circulation model. Geophys. Res. Lett..

[19] Konecky BL, Noone DC, Cobb KM (2019). The influence of competing hydroclimate processes on stable isotope ratios in tropical rainfall. Geophys. Res. Lett..

[20] Andersen KK (2006). The Greenland Ice Core Chronology 2005, 15–42ka. Part 1: constructing the time scale. Quat. Sci. Rev..

[21] Svensson A (2008). A 60 000 year Greenland stratigraphic ice core chronology. Climate.

[22] Wright HE (1983). Late-Pleistocene glaciation and climate around the Junín Plain, Central Peruvian Highlands. Geografiska Annaler. A Phys. Geogr..

[23] Smith JA, Finkel RC, Farber DL, Rodbell DT, Seltzer GO (2005). Moraine preservation and boulder erosion in the tropical Andes: interpreting old surface exposure ages in glaciated valleys. J. Quat. Sci..

[24] Smith JA, Seltzer GO, Farber DL, Rodbell DT, Finkel RC (2005). Early local last glacial maximum in the tropical Andes. Science.

[25] Sagredo EA, Rupper S, Lowell TV (2014). Sensitivities of the equilibrium line altitude to temperature and precipitation changes along the Andes. Quat. Res..

[26] Kaser G (2001). Glacier-climate interaction at low latitudes. J. Glaciol..

[27] Shakun JD (2015). Cosmogenic dating of Late Pleistocene glaciation, southern tropical Andes, Peru. J. Quat. Sci..

[28] Bakke J (2009). Rapid oceanic and atmospheric changes during the Younger Dryas cold period. Nat. Geosci..

[29] Rodbell DT, Seltzer GO, Mark BG, Smith JA, Abbott MB (2008). Clastic sediment flux to tropical Andean lakes: records of glaciation and soil erosion. Quat. Sci. Rev..

[30] Seltzer G, Rodbell D, Burns S (2000). Isotopic evidence for late Quaternary climatic change in tropical South America. Geology.

[31] Heyman J (2016). Boulder height—exposure age relationships from a global glacial 10Be compilation. Quat. Geochronol..

[32] Martin LCP (2015). In situ cosmogenic 10Be production rate in the High Tropical Andes. Quat. Geochronol..

[33] Kelly MA (2015). A locally calibrated, late glacial 10 Be production rate from a low-latitude, high-altitude site in the Peruvian Andes. Quat. Geochronol..

[34] Urrego DH, Bush MB, Silman MR (2010). A long history of cloud and forest migration from Lake Consuelo, Peru. Quat. Res..

[35] Wang X (2017). Hydroclimate changes across the Amazon lowlands over the past 45,000 years. Nature.

[36] Seltzer GO (2002). Early warming of tropical South America at the last glacial-interglacial transition. Science.

[37] Waelbroeck C (2002). Sea-level and deep water temperature changes derived from benthic foraminifera isotopic records. Quat. Sci. Rev..

[38] WAIS Divide Project Members et al. (2013). Onset of deglacial warming in West Antarctica driven by local orbital forcing. Nature.

[39] McGee D, Donohoe A, Marshall J, Ferreira D (2014). Changes in ITCZ location and cross-equatorial heat transport at the Last Glacial Maximum, Heinrich Stadial 1, and the mid-Holocene. Earth Planet. Sci. Lett..

[40] Lee J-Y, Wang B (2014). Future change of global monsoon in the CMIP5. Clim. Dyn..

[41] Broecker WS, Putnam AE (2013). Hydrologic impacts of past shifts of Earth’s thermal equator offer insight into those to be produced by fossil fuel CO_2_. Proc. Natl Acad. Sci. USA.

[42] Byrne MP, Pendergrass AG, Rapp AD, Wodzicki KR (2018). Response of the Intertropical convergence zone to climate change: location, width, and strength. Curr. Clim. Change Rep..

[43] Gregory, J. M. et al. A model intercomparison of changes in the Atlantic thermohaline circulation in response to increasing atmospheric CO_2_ concentration. *Geophys. Res. Lett.* **32**, L12703 (2005).

[44] Held IM, Soden BJ (2006). Robust responses of the hydrological cycle to global warming. J. Clim..

[45] Neelin JD, Munnich M, Su H, Meyerson JE, Holloway CE (2006). Tropical drying trends in global warming models and observations. Proc. Natl Acad. Sci. USA.

[46] Hatfield RG (2020). Stratigraphic correlation and splice generation for sediments recovered from a large-lake drilling project: an example from Lake Junín, Peru. J. Paleolimnol..

[47] Reimer PJ (2013). IntCal13 and Marine13 radiocarbon age calibration curves 0–50,000 years cal BP. Radiocarbon.

[48] Weninger B, Jöris O (2008). A 14C age calibration curve for the last 60 ka: the Greenland-Hulu U/Th timescale and its impact on understanding the Middle to Upper Paleolithic transition in Western Eurasia. J. Hum. Evol..

[49] Blaauw M, Christen JA (2011). Flexible paleoclimate age-depth models using an autoregressive gamma process. Bayesian Anal..

