# Supplementary material for: Andean drought and glacial retreat tied to Greenland warming during the last glacial period
Source: Nat Commun. 2020 Oct 12;11:5135. doi: 10.1038/s41467-020-19000-8 (PMC7552390; doi:10.1038/s41467-020-19000-8)
Supplement: Supplementary file 1 — Supplementary Information [file 41467_2020_19000_MOESM1_ESM.pdf]

Supplementary Information for

Andean drought and glacial retreat tied to Greenland warming during the last glacial period

Woods et al.

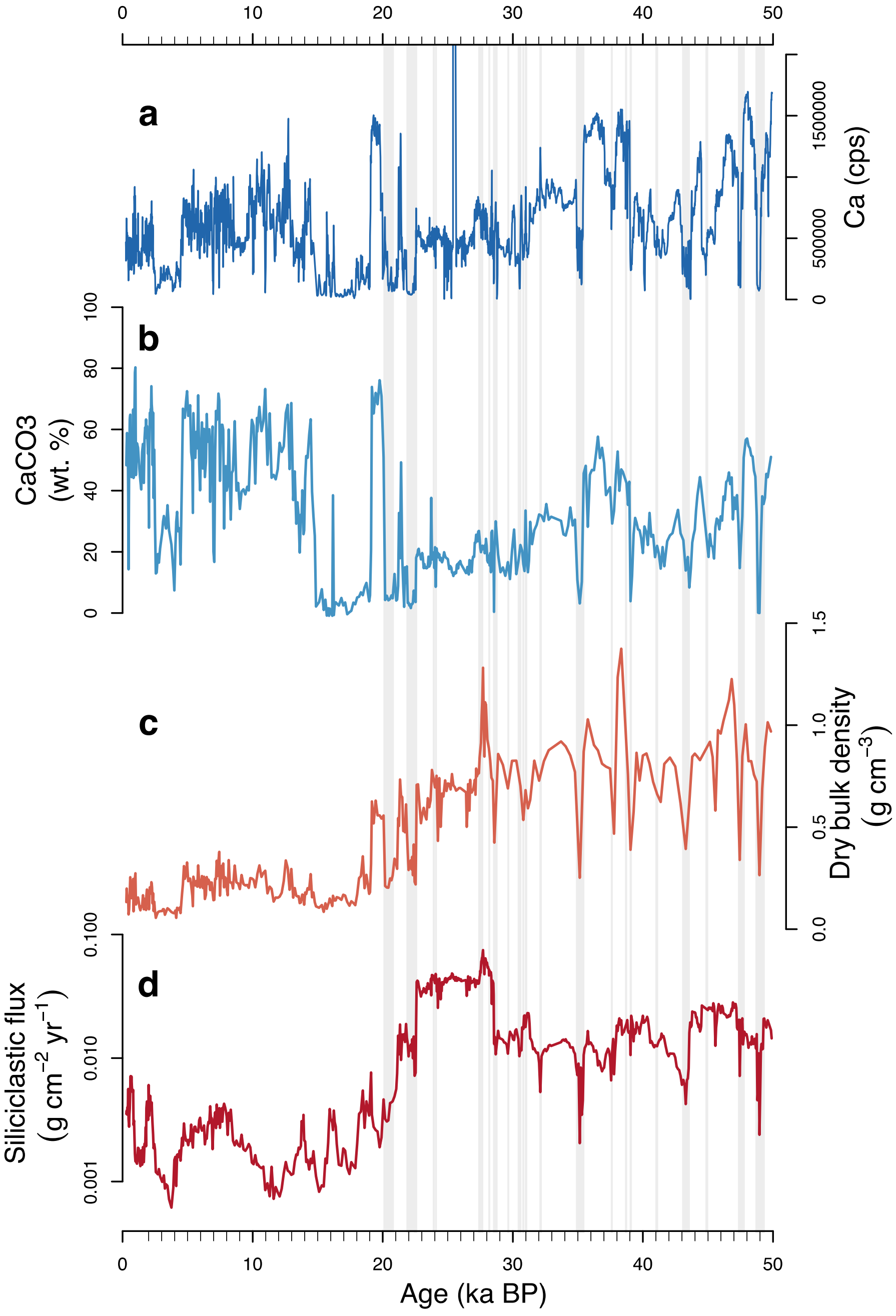

Supplementary Fig. 1 | The carbonate content of Junín sediments reflects different processes during glacial versus interglacial periods. Elevated $CaCO_3$ from 20-50 ka indicates glacially-generated detrital inputs derived from the extensive limestone bedrock in the valleys surrounding the lake. During the late-glacial and Holocene, when glaciers are no longer present in the watershed, authigenic precipitation of fine-grained calcite dominates the sedimentary record[1]. **a**, Calcium (cps) from scanning XRF closely resembles **b**, sedimentary $CaCO_3$ content (wt. %). **c**, dry bulk density. **d**, siliciclastic flux.

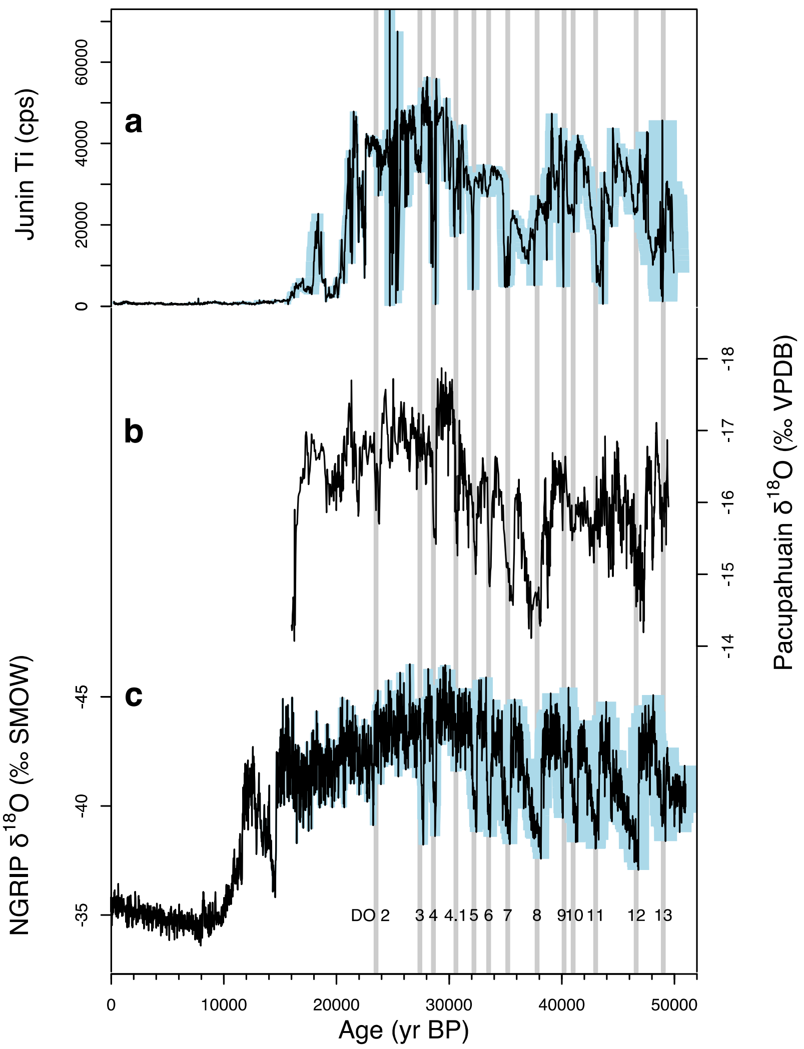

Supplementary Fig. 2 | The timing of Dansgaard-Oeschger (DO) interstadials, as recorded by decreases in glacigenic sediment flux to Lake Junín and increases in the $\delta^{18}O$ value of the NGRIP ice core record, overlap within the age model chronological uncertainties. Continuous error estimates are not available for the Pacupahuain speleothem time series, but the 2σ errors for individual ^{230}Th ages are small, ranging from ±40 to ±150 years. **a**, NGRIP $\delta^{18}O$ (*2*,*3*) based on the GICC05 timescale (black line) and the maximum counting error (blue shaded region), which should be regarded as 2σ as discussed in Andersen et al. (2006). **b**, Pacupahuain speleothem $\delta^{18}O$ (*4*). **c**, Lake Junín Ti counts per second (cps) (black line) and age model 95% confidence interval (blue shaded region) based on the Bacon age model discussed in the text. Numbered grey vertical lines indicate DO interstadials.

Supplementary Table 1 | Radiocarbon ages for the Junín cores

UCI AMS#	Core	Mean depth (cm)	Total depth (m)	Material	^{14}C age (BP)	±1σ	Median age (cal BP)	95% prob. lower range	95% prob. upper range
164796	C15-D1	6-7	0.215	charcoal	330	35	390	305	475
201049	C15-D1	50-51	0.655	charcoal	630	60	605	530	675
164797	C15-D2	16-17	0.98	charcoal	1815	20	1760	1710	1820
164798	C15-D3	16-19	1.47	charcoal	2275	25	2320	2170	2350
209665	C15-D3	23-24	1.61	charcoal	2510	35	2590	2490	2740
164802	D15-D3	16-19	1.875	charcoal	3440	25	3690	3620	3830
201050	D15-D3	19-21	1.9	charcoal	3900	80	4320	4090	4530
164803	D15-D3	39-42	2.105	charcoal	3990	45	4470	4300	4780
209668	D15-D3	44-45	2.145	charcoal	4060	70	4570	4420	4820
164799	C15-D6	9-12	2.83	charcoal	5775	25	6580	6500	6640
164800	C15-D6	56-59	3.3	charcoal	6915	30	7740	7680	7820
193150	1D-2H-2	10-13	3.43	charcoal	7310	90	8120	7970	8320
193151	1D-2H-2	21-22	3.53	charcoal	7645	50	8440	8380	8540
193101	D15-D6	41-42	3.805	plant macrofossil	8730	150	9790	9500	10200
201051	C15-D7	10-11	3.84	charcoal	9100	230	10250	9560	11050
201052	C15-D7	21-23	3.89	charcoal	9145	50	10300	10200	10500
201053	C15-D7	31-33	3.99	charcoal	9670	120	11000	10650	11200
209666	C15-D7	70-71	4.375	charcoal	11670	230	13500	13100	14050
209667	C15-D7	81-83	4.49	plant macrofossil	11940	70	13750	13600	14000
193144*	1C-2H-1	7-8	4.57	plant macrofossil	10850	150	12750	12550	13050
193098	C15-D7	90-91	4.575	plant macrofossil	11860	130	13700	13450	14000
209669	D15-D7-1	42-44	4.64	charcoal	12070	90	13900	13750	14150
201054	C15-D8	11-12.5	4.81	charcoal	12500	45	14750	14350	15058
172568	1C-2H-1	35-36	4.85	plant macrofossil	12605	35	15000	14750	15150
201055	C15-D8	16-17	4.855	plant macrofossil	12940	270	15450	14450	16250
193145	1C-2H-1	39-40	4.89	plant macrofossil	13000	200	15550	15000	16150
201056	C15-D8	21-22	4.905	plant macrofossil	13540	300	16350	15500	17250
193099	C15-D8	28-29	4.975	plant macrofossil	13200	40	15850	15700	16050
164801	C15-D8	51-54	5.215	plant macrofossil	13320	70	16000	15800	16250
193146	1C-2H-1	95-96	5.64	plant macrofossil	15670	80	18900	18750	19100
164804	D15-D8	41-44	5.665	plant macrofossil	15705	45	18950	18800	19100
193102	D15-D8	46-47	5.705	plant macrofossil	15740	120	19000	18750	19300
201057	D15-D8	48-50	5.73	plant macrofossil	15540	140	18800	18500	19100
193103	D15-D8	62-64	5.87	plant macrofossil	16550	220	19950	19500	20500
193104	D15-D8	65-66	5.895	plant macrofossil	16675	45	20100	19950	20300
201058	D15-D8	67-68	5.915	plant macrofossil	16150	420	19500	18650	20500
193147	1C-2H-1	127-128	5.95	plant macrofossil	16940	60	20450	20200	20600
193105	D15-D8	78-79	6.025	plant macrofossil	15420	60	18700	18550	18800
193106	D15-D8	83-84	6.075	plant macrofossil	17490	80	21100	20900	21400
193100	C15-D9	68-69	6.365	plant macrofossil	18100	60	21900	21700	22200
193148	1C-2H-2	19-20	6.375	plant macrofossil	18065	50	21900	21700	22100
164805	D15-D9	12-14	6.39	plant macrofossil	18055	45	21900	21700	22100
193149	1C-2H-2	44-45	6.61	plant macrofossil	18495	50	22400	22300	22500
164806	D15-D9	36-38	6.63	plant macrofossil	18660	60	22500	22400	22700
181187	1C-2H-2	47-48	6.64	plant macrofossil	18850	60	22700	22500	22900
193107*	D15-D9	41-42	6.675	plant macrofossil	13910	35	16850	16650	17050
201059	D15-D9	41-42	6.675	charcoal	18715	45	22600	22400	22700
181189	1B-4H-1	126-127	10.999	charcoal	24720	740	28900	27700	30500
193161	1B-4H-1	127-128	11.009	charcoal	24540	200	28600	28100	29000

201037	1B-4H-1	130-131	11.039	plant macrofossil	24120	100	28100	27900	28500
201038	1B-4H-1	130-131	11.039	charcoal	24210	80	28200	28000	28500
193162	1B-4H-1	143-145	11.174	plant macrofossil	23730	100	27800	27600	28000
181190	1D-5H-1	21-22	11.194	plant macrofossil	23680	110	27800	27600	28000
193163	1B-4H-2	16-17	11.429	charcoal	25680	170	29900	29400	30400
181191	1D-5H-1	48-49	11.444	plant macrofossil	25480	190	29600	29100	30200
193164	1B-4H-2	40-41	11.669	plant macrofossil	26550	170	30800	30500	31100
172616	1D-5H-1	78-79	11.749	plant macrofossil	27100	230	31100	30800	31400
181192	1B-4H-2	50-51	11.769	charcoal	25650	410	29800	29000	30700
193153	1D-5H-1	101-102	11.979	plant macrofossil	27090	140	31100	30900	31300
181193	1B-4H-2	91-93	12.184	plant macrofossil	28390	230	32300	31600	33000
181194	1D-5H-2	36-37	12.819	charcoal	31370	330	35300	34700	36000
193154	1D-5H-2	43-44	12.889	plant macrofossil	31460	230	35300	34800	35900
181195	1D-5H-2	102-103	13.479	plant macrofossil	34280	470	38800	37700	40000
193155*	1D-5H-2	102-103	13.479	plant macrofossil	27420	150	31300	31100	31500
193165	1B-5H-1	91-93	13.684	plant macrofossil	34320	730	38800	37000	40500
181196	1D-5H-2	134-135	13.799	charcoal	35050	430	39600	38700	40600
193166	1B-5H-1	110-113	13.919	charcoal	33460	430	37700	36600	38700
201039	1B-5H-1	113-114	13.939	charcoal	34620	250	39100	38600	39700
201040*	1B-5H-1	133-134	14.139	charcoal	32960	220	37000	36400	37900
181199*	1B-5H-1	136-139	14.179	plant macrofossil	17930	110	21700	21400	22000
193167	1B-5H-2	8-10	14.398	plant macrofossil	34330	490	38800	37700	40000
193156	1D-6H-1	51-54	14.578	plant macrofossil	36540	430	41100	40300	41900
201041	1B-5H-2	38-41	14.703	plant macrofossil	36440	310	41100	40400	41700
172569	1B-5H-2	71-72	15.023	plant macrofossil	39270	500	43100	42400	44000
181200	1B-5H-2	72-73	15.033	plant macrofossil	40460	840	44000	42900	45500
181201	1B-5H-2	72-73	15.033	plant macrofossil	39860	630	43600	42700	44700
181197	1D-6H-1	96-97	15.033	charcoal	40550	680	44100	43100	45300
181198	1D-6H-1	96-97	15.033	plant macrofossil	39800	1530	43600	41800	46600
201042	1B-5H-2	81-83	15.128	plant macrofossil	41070	560	44600	43600	45600
201043	1B-5H-2	83-84	15.143	plant macrofossil	40160	490	43800	43000	44700
201044	1B-5H-2	108-111	15.403	plant macrofossil	40560	520	44100	43200	45100
193157	1D-6H-1	147-148	15.538	plant macrofossil	40930	730	44400	43300	45700
193158*	1D-6H-2	8-11	15.683	plant macrofossil	35780	570	40400	39300	41500
193159*	1D-6H-2	34-36	15.938	plant macrofossil	18810	70	22700	22500	22900
193160*	1D-6H-2	107-110	16.665	plant macrofossil	41200	1300	44700	42800	47400
201045*	1B-6H-1	111-112	17.145	charcoal	49800	1600	55500	50200	57300
201046	1B-6H-1	129-130	17.325	charcoal	46700	1500	50300	46800	53400
201047	1B-6H-2	7-10	17.574	plant macrofossil	47400	1600	51300	47300	54500
193168	1B-6H-2	8-9	17.574	plant macrofossil	52600	3000	–	–	–
201048*	1B-6H-2	10-11	17.594	plant macrofossil	36640	730	41100	39900	42300
193169*	1B-6H-2	44-45	17.934	plant macrofossil	43300	960	46600	45100	48800
172570	1D-7H-1	82-84	17.994	plant macrofossil	>50800	–	–	–	–
172617	1D-7H-2	134-136	20.014	charcoal	>53400	–	–	–	–

Supplementary References

1. Seltzer, G., Rodbell, D. & Burns, S. Isotopic evidence for late Quaternary climatic change in tropical South America. *Geology* **28**, 35–38 (2000).

2. Andersen, K. K. *et al.* The Greenland Ice Core Chronology 2005, 15–42ka. Part 1: constructing the time scale. *Quatern. Sci. Rev.* **25**, 3246–3257 (2006).
3. Svensson, A. *et al.* A 60 000 year Greenland stratigraphic ice core chronology. *Clim. Past* **4**, 47–57 (2008).
4. Kanner, L. C., Burns, S. J., Cheng, H. & Edwards, R. L. High-Latitude Forcing of the South American Summer Monsoon During the Last Glacial. *Science* **335**, 570–573 (2012).
